# Efficacy of PARP inhibition in *Pde6a* mutant mouse models for retinitis pigmentosa depends on the quality and composition of individual human mutations

**DOI:** 10.1038/cddiscovery.2016.40

**Published:** 2016-07-04

**Authors:** K Jiao, A Sahaboglu, E Zrenner, M Ueffing, P A R Ekström, F Paquet-Durand

**Affiliations:** 1 Cell Death Mechanisms Group, Division of Experimental Ophthalmology, Centre for Ophthalmology, Institute for Ophthalmic Research, University of Tuebingen, Roentgenweg 11, Tuebingen 72076, Germany; 2 Centre for Ophthalmology, The Second People’s Hospital of Yunnan Province and The Fourth Affiliated Hospital of Kunming Medical University, Qingnian 176, Kunming 650021, China; 3 Centre for Ophthalmology, Institute for Ophthalmic Research, University of Tuebingen, Tuebingen 72076, Germany; 4 Werner Reichardt Centre for Integrative Neuroscience (CIN), University of Tuebingen, Tuebingen, Germany; 5 Division of Ophthalmology, Department of Clinical Sciences, Lund, University of Lund, Lund 22184, Sweden

## Abstract

Retinitis pigmentosa (RP), an inherited blinding disease, is caused by a variety of different mutations that affect retinal photoreceptor function and survival. So far there is neither effective treatment nor cure. We have previously shown that poly(ADP-ribose)polymerase (PARP) acts as a common and critical denominator of cell death in photoreceptors, qualifying it as a potential target for future therapeutic intervention. A significant fraction of RP-causing mutations affect the genes for the rod photoreceptor phosphodiesterase 6A (PDE6A) subunit, but it is not known whether they all engage the same death pathway. Analysing three homozygous point mutations (*Pde6a* R562W, D670G, and V685M) and one compound heterozygous *Pde6a*^*V685M*/*R562W*^ mutation in mouse models that match human RP patients, we demonstrate excessive activation of PARP, which correlated in time with the progression of photoreceptor degeneration. The causal involvement of PARP activity in the neurodegenerative process was confirmed in organotypic retinal explant cultures treated with the PARP-selective inhibitor PJ34, using different treatment time-points and durations. Remarkably, the neuroprotective efficacy of PARP inhibition correlated inversely with the strength of the genetically induced insult, with the D670G mutant showing the best treatment effects. Our results highlight PARP as a target for neuroprotective interventions in RP caused by *PDE6A* mutations and are a first attempt towards personalized, genotype-matched therapy development for RP. In addition, for each of the different mutant situations, our work identifies windows of opportunity for an optimal treatment regimen for further *in vivo* experimentation and possibly clinical studies.

## Introduction

Retinitis pigmentosa (RP) is a hereditary disease that causes the progressive degeneration and death of photoreceptors and is one of the main causes of blindness in the developed world,^1^ affecting ~1 in 4000 people.^2^ RP is genetically and clinically heterogeneous, with onset varying from early childhood to late adulthood depending on the exact mutation and, probably, other factors. Mutations in more than 60 genes are associated with RP (https://sph.uth.edu/retnet), giving a complexity that may require personalized therapy approaches, which in turn necessitates knowledge on the pathology of the individual mutations. At the same time, there is a need to identify common principles that can be exploited for the development of therapies addressing many different types of mutations at once.

As many as 10% of human RP patients may suffer from mutations in one of the three genes encoding for phosphodiesterase 6 (PDE6).^3–5^ In rod photoreceptors, PDE6 consists of two catalytic subunits *α* and *β* (A and B) and an inhibitory *γ* (G) subunit and has the function to hydrolyse cGMP in response to light. Although each of the three PDE6 subunits can be affected by mutations, previous research on *Pde6* mutant animals has mostly focussed on *Pde6b* mutants, namely the *rd1* and *rd10* mouse models.^6,7^ An excessive activation of poly(ADP-ribose)polymerase (PARP) has been shown to not only have an important role in many neurodegenerative diseases but it may also contribute to caspase-independent photoreceptor cell death.^8,9^ Interestingly, in the *Pde6b* mutants, PARP activity was shown to be involved in the progression of photoreceptor degeneration, and PARP appears also to directly participate in photoreceptor degeneration in models with unrelated mutations.^10,11^ PARP therefore has the quality of a common denominator in photoreceptor degeneration, and is, as such, an interesting molecular target, although mutation-dependent aspects may very well occur.^11^ To date, *Pde6a* mutants have been far less studied and it is not clear if they adhere to such a PARP involvement, nor if the possible characteristics of the latter will make it available for neuroprotective intervention.

At least 29 different *PDE6A* mutations are known from RP patients (the human gene mutation database; http://www.hgmd.cf.ac.uk; information retrieved July 2015); thus, to understand the degenerative events in the human situation, it is necessary to come as close as possible to the defined genetic defects when we select our study material. Here, we used three different homozygous *Pde6a* mutant mice, *Pde6a*^*R562W*^, *Pde6a*^*D670G*^, and *Pde6a*^*V685M*^, as well as compound heterozygous *Pde6a*^V685M*R562W^ animals. Importantly, genotypes homologous to the homozygous *Pde6a*^V685M/V685M^ and the compound heterozygous *Pde6a*^V685M*R562W^ mouse mutant, respectively, are present among RP patients.^12,13^ This therefore provided us with the rare opportunity to attempt a personalized medicine approach, studying two RP mouse models genotype-matched to human RP, both with respect to the mechanistic components and to how they respond to a defined treatment. The two additional mutations further increased the insight into how alternative mutations in the same gene can affect such parameters. For the sake of brevity, in the following, we refer to the *Pde6a* mutant animals as V685M, V685M*R562W, R562W, and D670G, respectively.

Using these homozygous and compound heterozygous *Pde6a* mutant animals, we show that PARP activity during photoreceptor neurodegeneration is raised upon *Pde6a* deficiency. Conversely, *in vitro* based tests indicate that the selective inhibition of PARP reduces photoreceptor cell death, an effect that was found dependent on the severity of the genetically induced insult and the time-point of therapeutic intervention. Our study thus suggests the likelihood of individual onset and progression patterns in human RP patients associated with different mutations. The use of rodent disease models, matched to individual patients, can therefore support preclinical pharmacological therapy development for RP that reflects the genetic heterogeneity in the human condition.

## Results

### PARP activity is increased in *Pde6a* mutants, concomitant with cell death

**Figure 1:**
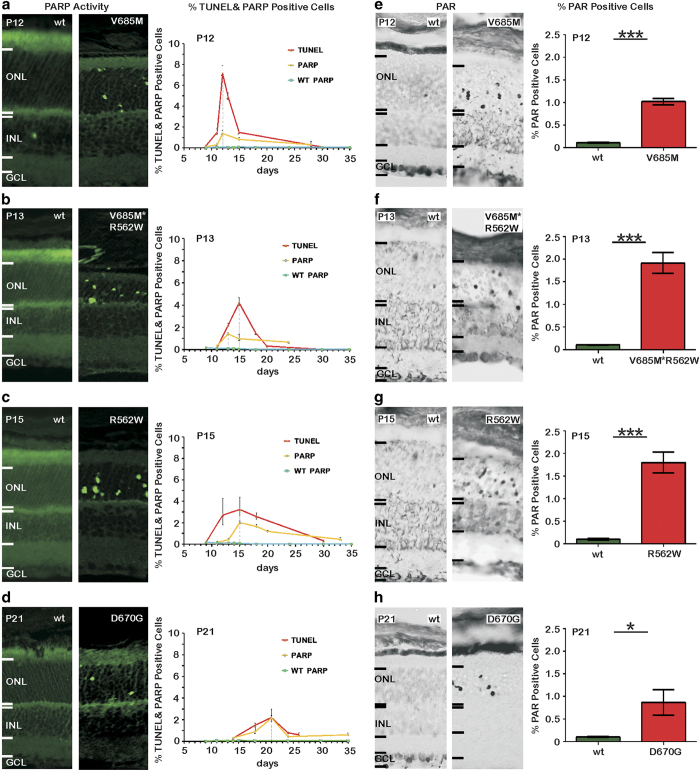
PARP activity and PARylation in *Pde6a* mutant photoreceptors. The number of PARP activity-positive cells in the V685M, V685M*R562W, R562W, and D670G photoreceptors was strongly increased when compared with wt cells. The quantification of PARP activity-positive cells during the first 30 postnatal days (orange curve) identified peaks of activity at P12, P13, P15, and P21 in the V685M, V685M*R562W, R562W, and D670G mutants, respectively. Asterisks indicate significant levels of mutant *versus* wt PARP activity. A comparison with the number of dying, TUNEL-positive cells (red curve) in three out of four mutations showed a strong correlation with the peaks of PARP activity, while in the V685M*R562W model, PARP activity appeared to precede cell death (**a**–**d**). Similarly, a staining for the accumulation of PARylated proteins – products of PARP activity – showed a strong increase in all four *Pde6a* mutants when compared with wt cells. At the respective peaks of PARP activity, the number of PAR-positive cells was significantly increased in all four mutants (**e**–**h**). The images shown are representative for observations on at least six different specimens for each genotype. Values shown in line and bar graphs are mean±S.E.M., with *n*=6 in all cases. For easy reference, the peak time-points of PARP activity are stated in each such graph (**a**–**d**).

Previous studies showed that PARP activity and the accumulation of its product poly(ADP-ribose) was increased during photoreceptor degeneration in the *Pde6b* mutant *rd1* mouse retina.^10^ Hereafter, we refer to poly(ADP-ribose) as PAR and to the process as PARylation. We first performed an *in situ* PARP activity assay to analyse the temporal appearance of PARP activity in the photoreceptor layer in *Pde6a* mutants. The number of photoreceptor cells showing high PARP activity was quantified and peak activities were found at P12, P13, P15, and P21 for the V685M, V685M*R562W, R562W, and D670G mutants, respectively (Figures 1a–d).

At the respective peaks of PARP activity, a statistical analysis was performed, and in all *Pde6a* mutant genotypes the number of PARP activity-positive cells was significantly higher compared with that in the corresponding wild-type (wt) cells (Figures 1a–d). Previously, we had assessed the progression of photoreceptor degeneration in the various *Pde6a* mutants, using the terminal deoxynucleotidyl transferase dUTP nick-end labelling (TUNEL) assay to label dying cells in the outer nuclear layer (ONL) at different postnatal ages.^13^ These data showed peaks of cell death at P12, P15, P15, and P21 for the V685M, V685M*R562W, R562W, and D670G mutants, respectively, and is reproduced here for comparison only. We consider the occurrence of the peak of cell death to correlate with the progression of retinal degeneration such that the V685M mutant with its early peak is the most rapidly degenerating model of the four.

In all these situations, the peak of PARP activity in principle coincided in time with the peak of photoreceptor cell death. The relative proportion of cells showing PARP activity (i.e. the peak height) was, however, different in the various genotypes. In the very rapidly degenerating V685M mutant, at the peak of cell death, only ~1.4% of cells displayed high PARP activity. This corresponded to 20% of dying cells. In the more slowly degenerating V685M*R562W and R562W mutants, the proportion of PARP activity-positive cells *versus* dying cells was higher, whereas in the D670G mutant, with the slowest progression of retinal degeneration, the number of cells showing high PARP activity was essentially the same (i.e. 100%) as the number of dying cells.

Excessive PARP activity may lead to an accumulation of its product PAR, which can be studied using immunohistochemistry.^14^ This staining suggested increased PARylation in the photoreceptor layer in each *Pde6a* mutant (Figures 1e–h). Quantification and statistical analysis demonstrated significant increases in the number of PAR-positive cells in all *Pde6a* mutants at the respective peak of degeneration, providing an independent confirmation of the excessive activation of PARP in dying photoreceptors.

### PARP inhibition delays photoreceptor cell death in PDE6a mutants

The number of TUNEL- and PARP-positive cells in the ONL of *Pde6a* mutants was strongly increased when compared with wt cells. In addition, the appearance of cell death and PARP activity was highly correlated in time, suggesting a causal relationship between the two. As inhibition of PARP can protect the photoreceptors of the cultured *rd1* retina,^10^ we hypothesized that *Pde6a* mutants would likewise be helped by such intervention.

Retinal explants from *Pde6a* mutants were cultured and treated with 6 *μ*M PJ34, a well-known inhibitor of PARP that is effective on degenerating photoreceptors at this concentration.^10^ To reveal the effective treatment period for neuroprotection, we tested this compound in both short- and long-term approaches. First, we applied a short-term culture paradigm, which consisted of starting the cultures when the mice were at P5 and finishing them when they were at a stage corresponding to P15. Following 4 days *in vitro* (DIV) of adaptation, the cultures were treated with PJ34, from P9 until P15 (i.e. P5+4+6 DIV; see Material and Methods), after which the TUNEL assay was used to quantitate photoreceptor cell death. In addition, counting the number of ONL cell rows was used to assess the photoreceptor survival.

**Figure 2:**
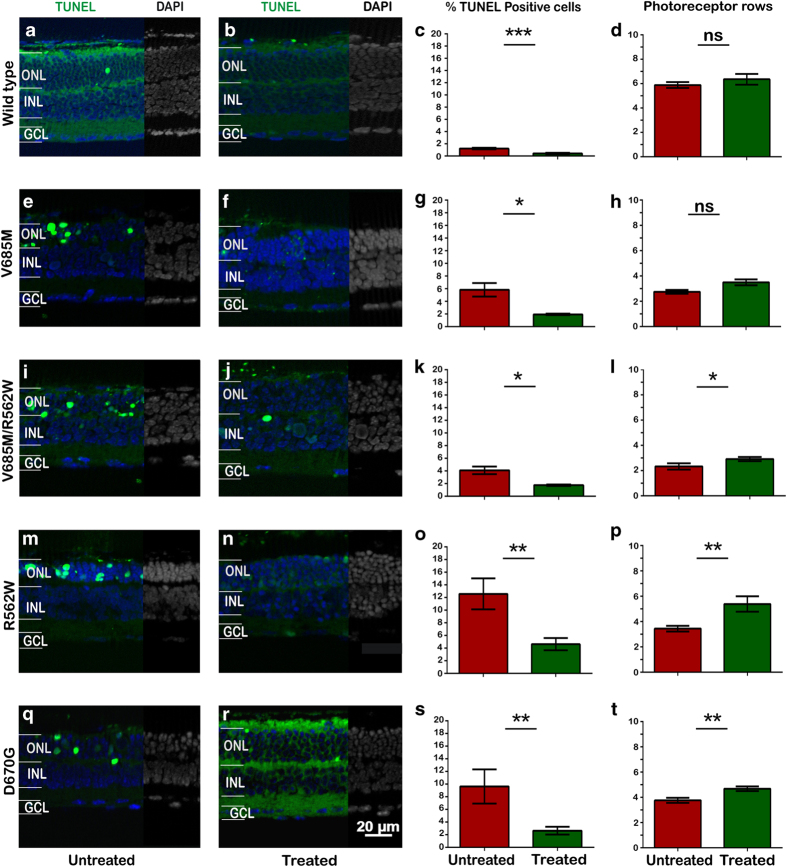
PARP inhibition delays photoreceptor degeneration in *Pde6a* mutants. In short-term retinal explant cultures (P5*-*P15; i.e. P5+10 DIV, of which the last 6 days were with treatment), the number of dying, TUNEL-positive cells (green) in the ONL of wt retina (**a** and **b**) was significantly reduced by treatment with PJ34 (**c**), whereas the number of surviving photoreceptor rows remained unchanged (**d**). In the rapidly degenerating V685M mutant, at P15, PJ34 reduced the amount of cell death without significantly increasing photoreceptor survival (**e**–**h**). In the more slowly degenerating V685M*R562W (**i**–**l**), R562W (**m**–**p**), and D670G retinas (**q**–**u**), a reduction of cell death lead to significant increases in photoreceptor survival. This prosurvival effect was most pronounced in the slowest degenerating mutants R562W and D670G. Images shown are representative for at least six different specimens for each genotype; DAPI (4',6-diamidino-2-phenylindole; blue/grey) was used as a nuclear counterstain.

All *Pde6a* mutants demonstrated high levels of TUNEL positivity in the untreated groups (Figure 2). However, PARP inhibition with PJ34 significantly decreased the percentages of dying photoreceptors in all four *Pde6a* mutants (Figure 2), suggesting that these mutants responded to PARP inhibition in a favourable way. Interestingly, even though the basal TUNEL value for untreated wt explants was low (about 1%), treatment with PJ34 was still able to reduce this in a significant manner, indicating that also the stress evoked by the explantation and culturing lead the photoreceptors into a PARP-dependent death pathway. Conversely, when the treatment effects were analysed as the number of surviving rows of photoreceptors, rather than the number of dying cells, there was a very clear indication that the TUNEL reduction translated into increased photoreceptor survival, as all treated mutant explants had an average cell row number that was higher compared with that in their untreated counterparts. Moreover, even within the short time frame of this paradigm (treatment from P9 to P15), PJ34 treatment resulted in significantly higher values for surviving cell rows in V685M*R562W, R562W, and D670G explants (Figure 2).

### PARP inhibition decreases PAR accumulation in *Pde6a* mutant retina

**Figure 3:**
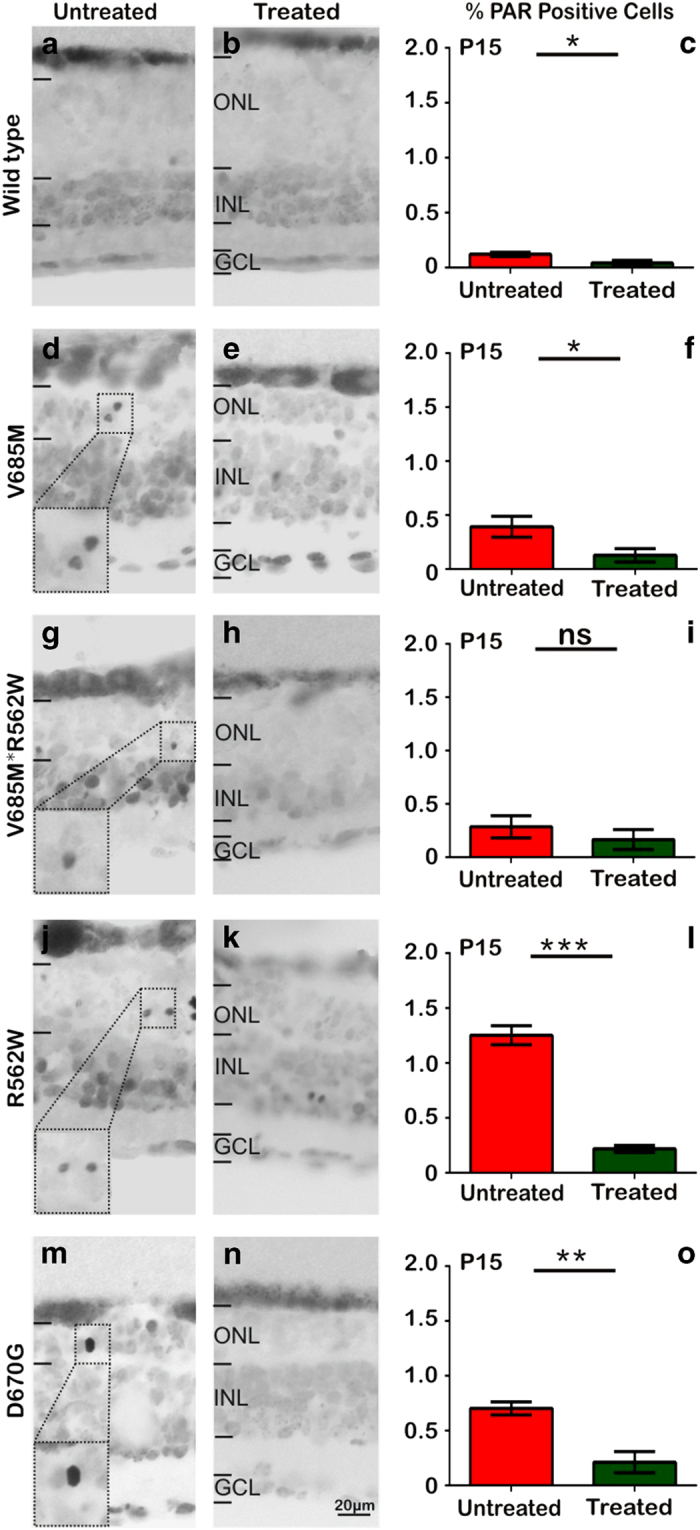
PARP inhibition decreases PAR accumulation in *Pde6a* mutant retina. In photoreceptors excessive PARP activity results in the accumulation of PAR. In retinal explant cultures, at P15, PAR-positive cells were seen occasionally in the wt situation (**a**), yet with PJ34 treatment, resulting in a significant decrease of their numbers (**b**, quantification in **c**). A similar effect of PARP inhibition was seen in the V685M mutant (**d**–**f**), whereas in the V685M*R562W the reduction of PAR-positive cells did not attain statistical significance (**g**–**i**). The most pronounced effects of PARP inhibition were seen in the slowly degenerating R562W (**j**–**l**) and D670G (**m**–**o**) mutants. Images shown are representative of six different specimens per genotype.

To confirm that the short-term effects of PJ34 were indeed related to PARP inhibition, we analysed if the PJ34 treatment led to any reduced PARP activity as expressed by the formation of PAR polymers, or PARylation. As shown in Figure 1, PAR-positive cells were identified in the ONL of all *Pde6a* mutants and the number of PAR-positive cells was clearly reduced by PJ34 treatment (Figure 3). Although this effect was visible even in wt explant cultures, the reduction of ONL cells displaying PAR accumulation was most pronounced in the more slowly degenerating R562W and D670G mutants. These results thus indicated that PJ34 treatment indeed had strongly reduced the PARP activity in retinal photoreceptors.

### Long-term retinal explant cultures delineate windows of opportunity

When the culturing period was prolonged from P15 to P19, and thus the treatment extended to 10 DIV (P9–P19), PJ34 replicated very well its actions in the shorter term. As described previously, PJ34 had a significant positive effect on the number of surviving photoreceptor rows in all models except the V685M mutant (Supplementary Figure S1). In fact, the effects as such may even be seen as proportionally higher with the longer treatment, at least for the V685M*R562W and D670G mutants, since in these PJ34 now about doubled the number of remaining rows. We also note that in the untreated V685M, V685M*R562W, and R562W mutants, the number of residual photoreceptor rows was decreased to ~0.5, indicating that at this stage likely only cone photoreceptors were remaining.

Further prolongation of the culture duration to P25 changed the picture considerably, to the point that no significant photoreceptor rescue could be detected in any of the four *Pde6a* mutants (Supplementary Figure S2). Only in the slowest degenerating D670G mutant did PJ34 treatment result in a minor, but statistically nonsignificant increase of photoreceptor survival.

**Figure 4:**
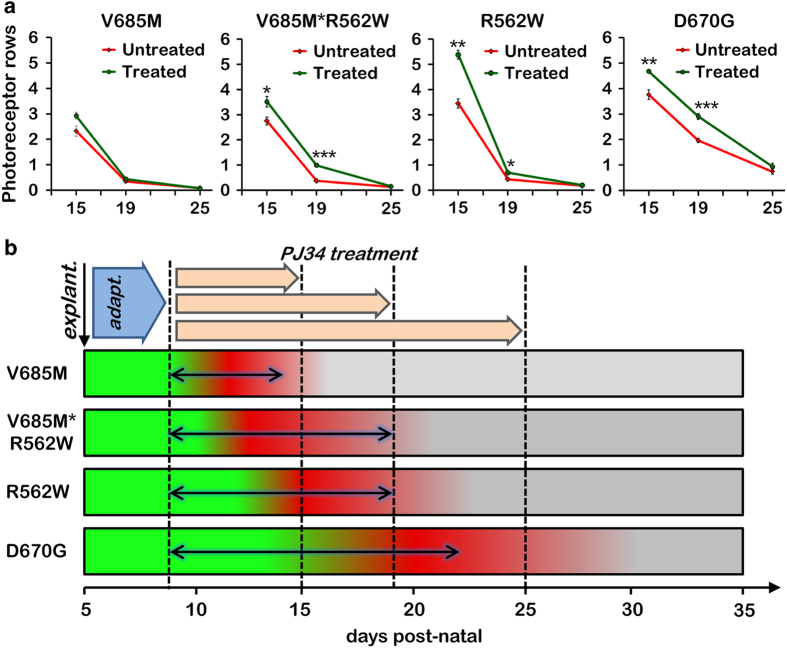
Treatment effects and windows of opportunity in different *Pde6a* mutants. When the number of surviving photoreceptor rows was plotted against the treatment duration, in the different genotypes, the effects of PJ34 treatment were variable with the V685M showing the smallest and the D670G mutant the biggest effect on photoreceptor survival (**a**). Retinas from *Pde6a* mutant animals were explanted at P5, left untreated to adapt to culture conditions for 4 days, and then treated with PJ34 until P15, P19, or P25. Initially, retinas appeared morphologically normal, healthy (illustrated here in green). During the degeneration period (red), ONL cells died until (virtually) all rod photoreceptors were lost (grey). The differences in treatment effects in *Pde6a* mutants corresponded with the onsets and speed of retinal degeneration, with V685M displaying the fastest and D670G the slowest disease progression. This resulted in differently sized windows of opportunity (black, double-headed arrows) with the slowest degenerating mutants displaying the largest window of opportunity (**b**).

This leaves us with a situation where the mutation-dependent rate of degeneration is reflected in the treatment outcomes. The most rapid degeneration (in the V685M model) could be treated to achieve an effect on TUNEL positivity in the short-term, 6 DIV treatment, but nothing else. The two medium rate degenerations (V685M*R562W, R562W) could be protected to the point of increased photoreceptor survival at both 6 and 10 DIV treatments, whereas the slowest degeneration (D670G) did the same, but in addition was the only one displaying a discernible numerical increase in surviving photoreceptor rows after treatment for 16 DIV. These results are summarized in Figure 4a, in which the treatment effects on photoreceptor survival are plotted against the treatment duration in the four *Pde6a* mutant genotypes. In this condensed view, the treatment effects are exemplified by the areas between the curves representing treated and untreated situations.

Taken together, the results thus suggest that for each mutant situation there was a specific window of opportunity for successful treatment, the size of which depended on the strength of the genetic insult (Figure 4b).

## Discussion

To date, RP is still an untreatable condition. Major obstacles for the successful personalized therapy development are the insufficient understanding of cellular disease mechanisms and the enormous heterogeneity of disease-causing mutations. Our study addresses these two problems by showing that PARP activity is a common denominator in retinal degeneration caused by three different point mutations in the *Pde6a* gene. In addition to studying the effects of PARP inhibition on these three mutations in homozygous situations, we also investigated a recently generated compound heterozygous model, which is genotype-matched to a human subject suffering from RP. We show that in all four mutant situations PARP inhibition affords a significant photoreceptor protection, with the effect size determined by the time-point of therapeutic intervention and the severity of the genetically induced insult.

### PDE6A mutations and cGMP signalling in retinitis pigmentosa

Human RP is characterized by a remarkable non-allelic genetic heterogeneity with around 60 different disease genes currently known (https://sph.uth.edu/Retnet; information retrieved in July 2015). Moreover, for each of these genes there are typically a large number of different disease-causing mutations, as is also true for the *PDE6A* gene for which at least 29 different mutations are known (http://www.hgmd.cf.ac.uk; information retrieved July 2015). In our attempts to create therapies that could act broadly on the various *PDE6A* RP genotypes, it is required that we increase our knowledge on if and how the degeneration processes differ between the latter. Several different mouse models carrying human homologous of *Pde6a* variants are known.^13,15^ In the present study, we took advantage of this and can now present mechanistic data on four such *Pde6a* mutation models, of which three are homozygous (V685M, R562W, and D670G), with the fourth being compound heterozygous (V685M*R562W).

Interestingly, the R562W, D670G, and V685M mutations all map to the catalytic domain of PDE6A.^15^ A previous study identified marked differences both in the expression of the different PDE6A mutant proteins and in the accumulation of cGMP, likely as a result of different consequences for the catalytic activity. In this respect, the V685M mutant showed the highest degree of cGMP accumulation, whereas the V685M*R562W and the R562W were intermediate, and the D670G showed a comparatively weak cGMP accumulation.^13^ This variation in the strength of the genetic insults likely is the reason for the different disease progression phenotypes.

### PARP activity in retinal degeneration

The activation of PARP is an important event during base excision repair of damaged DNA and PARP is sometimes addressed as the ‘guardian of the genome’.^16^ Accordingly, PARP activity was found to have prosurvival effects in the inner ear,^17^ and, in mice, the absence of PARP reduces the overall lifespan.^18^ Furthermore, for modern cancer therapy PARP inhibition is proposed as an adjuvant to increase the sensitivity of cancer cells to genotoxic stress.^19^ PARP is also involved in the epigenetic regulation of gene expression via the PARylation of histone proteins,^20^ and possibly also via modifications on the DNA methylation pattern.^21^ Taken together, these actions of PARP might explain the extensive changes in DNA methylation^22^ and gene expression^23^ observed during photoreceptor degeneration.

An excessive activation of PARP is also frequently observed during cell death, including in neurodegenerative diseases.^9,24,25^ When we used an *in situ* PARP activity assay based on the incorporation of biotin-labelled NAD^+^,^26^ we found that PARP activity was strongly increased in the photoreceptors of all four *Pde6a* mutant genotypes. This corresponds to earlier findings in the *Pde6b* mutant *rd1* and *rd10* mouse models for RP,^10,11^ and is also seen in many other animal models for RP, carrying disease-causing mutations in a variety of different genes.^11,27^ As most of these models also display increased photoreceptor cGMP, it is possible that this rise is a component of the PARP activation. How this would occur is not fully understood yet, although previous works have suggested a sequential activation of protein kinase G^28^ and histone deacetylase just before PARP activation.^29^

How could excessive PARP activity cause photoreceptor cell death? One possibility is that the excessive PARP-induced consumption of NAD^+^ leads to a depletion of energy-containing substrates, such as ATP, and thus causes an energetic collapse. An alternative possibility might be that excessive accumulation of certain PAR species could be toxic to the cell.^30^ In this context, it is interesting to note that the TUNEL assay marks cells that have already undergone a strong fragmentation of the DNA.^31,32^ The fact that PARP activity and TUNEL assays partly colocalize in the same photoreceptor cells^10^ thus suggests that excessive PARP activation may have contributed to DNA damage. We noted that the proportions of PARP activity- and TUNEL-positive cells differed between the models (Figure 1), in that during rapid degeneration there was a much higher percentage of TUNEL-positive compared with that in PARP activity-positive cells, whereas during slow degeneration the percentages were about equal. Whether this indicates a situation-dependent difference in execution times of the two processes remains to be studied.

### PARP inhibition as a therapeutic strategy to prevent RP

Although PARP activity and its role in DNA repair in general is seen as beneficial,^18^ in postmitotic neurons the demand for DNA repair is much lower, and hence under normal, physiological conditions, PARP inhibition or even genetic deletion has essentially no effect.^9,33^ However, under pathophysiological conditions, the aberrant and excessive PARP activation can be rectified by PARP inhibition, which would propose suitability in the RP situation.

The fact that PARP activity and also the accumulation of PAR colocalize with TUNEL^10^ may indicate that in the cascade of events leading to photoreceptor cell death, PARP activity is a relatively late event. While in general terms the targeting of late events may be less advantageous, in the case of PARP, the fact that many different mutations all cause PARP activity^11^ suggests that different cell death processes may converge on PARP as something like a late-stage common denominator. This makes PARP an attractive target for therapeutic interventions, as in diseases with a very high genetic heterogeneity, such as RP, this kind of therapy would be applicable to many different disease forms at once.

In the fastest degenerating V685M mutant, PJ34 treatment achieved only a relatively low degree of photoreceptor neuroprotection at P15, yet in the slowest degenerating D670G mutant, PJ34 treatment increased photoreceptor survival even at P25. Hence, in the different *Pde6a* mutants studied here the time window for therapeutic interventions was dependent on the strength of the genetically induced insult (Figure 4b). This provides a strong case for a personalized therapeutic approach, where the exact mutation will convey information with respect to how long the time window is open for PARP-centred interventions, and perhaps also other types of treatments.

Even when the correct window of opportunity was chosen (i.e. a treatment time-point and duration that showed an effect), PJ34 treatment did not halt retinal degeneration completely, in any of the four *Pde6a* mutants. While this could be due to insufficient inhibitory potential of PJ34 or even off-target effects,^34^ it could also be due to the concurrent activation of PARP-independent cell death pathways.^11,35^ In the latter scenario, the blocking of PARP activity would slow down the degeneration but could not ultimately prevent it. However, it is worth mentioning that even a comparatively minor delay in the progression of retinal degeneration in a rodent could potentially translate to many years of useful vision in human RP patients.^36^ In the current situation, where no treatment is available, this would already constitute a major progress in the development of medicines for rare diseases.

## Conclusion

This is the first report demonstrating an excessive activation of PARP in photoreceptors suffering from different point mutations in the *Pde6a* gene. Taken together with earlier data obtained in several other RP animal models,^11^ this adds further weight to the notion that PARP is a common denominator in photoreceptor cell death and hence a promising target for therapy development.

Homozygous V685M and compound heterozygous V685M/R562W *Pde6a* mutants used in this study were genotype-matched to specific forms of human *PDE6A* RP. Our study suggests that the strength of the insult caused by these mutations correlates with onset and progression of disease. This likely bears implications for any future personalized therapeutic intervention as the severity and differential character (homozygous *versus* compound heterozygous) of a mutation may affect efficacy of treatment, highlighting the need to identify the right treatment durations, analysis time-points, and animal models for future genotype-matched therapy.

In conjunction with a careful clinical assessment of human RP phenotypes, our observations may impact the future design of clinical studies; for instance, in helping to define which genotype to include, at what time-point in the disease course, and what magnitude of treatment effect to expect.

## Materials and Methods

### Experimental animals

C3H wt mice^37^ were obtained from the Tuebingen university in-house animal facility. Homozygous mice carrying the V685M (nmf282) and D670G (nmf363) mutations were obtained from Jackson Labs (Bar Harbor, MA, USA).^15^ The R562W mutant was generated recently by GenOway (Lyon, France).^13^ Compound heterozygous V685M*R562W mutants were generated at the Tuebingen University by cross-breeding the respective homozygous mutants.

Animals had free access to food and water, were housed under standard 12 h lighting conditions, and were used irrespective of gender. *Pde6a* mice were killed at different time-points for histology (P9–P35) or for retinal explant culture (P5). All procedures were approved by the Tuebingen University committee on animal protection and performed in compliance with the ARVO statement for the use of animals in Ophthalmic and Visual Research. Protocols compliant with Section 4 of the German law on animal protection were reviewed and approved by the 'Einrichtung fur Tierschutz, Tierarztlichen Dienst und Labortierkunde' (Notifications 16.06.12 and 01.06.14). All efforts were made to minimize the number of animals used and their suffering.

### Retinal explant cultures

Mice were killed using decapitation at P5, and their heads were cleaned with 70% ethanol. The eyes were removed under sterile conditions, and subsequently incubated in 0.12% Proteinase K at 37 °C for 5 min. They were then washed with basal R16 medium containing 10% FCS for 2–3 min to inactivate Proteinase K. After that, the cornea, sclera, and lens were removed, so that only neural retina and retinal pigment epithelium (RPE) remained. The retinas were cut into four wedges and transferred onto the culturing membrane (Millicell, no. PIHA03050; Millipore, Cork, Ireland) with the RPE facing the membrane. The retinas were cultured in R16 medium with supplements^38^ for 4 days without treatment to adapt to *in vitro* conditions. From P9 onwards they were treated with 6 *μ*M PJ34 until P15 (6 days of treatment), P19 (10 days of treatment), or P25 (16 days of treatment). During the culturing period, the R16 medium was changed every 2 days and cultures were terminated using 4% paraformaldehyde (PFA; Polysciences, Warrington, PA, USA) in 0.1 M phosphate buffer (pH 7.4) for 30 min at room temperature.

### Retinal tissue preparations and TUNEL assay

The animals were killed by decapitation; the eyes were immediately enucleated, and directly embedded in Tissue-Tek cryomatrix (Leica, Bensheim, Germany) to obtain unfixed, frozen tissue for later use in the PARP activity assay.

To obtain fixed retinal preparations for PAR immunohistochemistry, the eyes were fixed with 4% PFA in 0.1 M phosphate buffer (pH 7.4) for 30 min at room temperature. After fixation, eyes were washed with PBS for 10 min and cryoprotected by incubation in graded sucrose solutions (10%, 20%, and 30%). Subsequently, tissues were embedded in cryomatrix and vertical sections (12 *μ*m) were obtained on a Leica CM3050S Microtome (Leica Biosystems, Wetzlar, Germany), air dried at 37 °C for 1 h, and stored at −20 °C until use.

The TUNEL Cell Death Detection Kit (conjugated with either fluorescein or TMR; Roche Diagnostics GmbH, Mannheim, Germany) was used to detect photoreceptor cell death, and performed according to the manufacturer’s instructions.

### PARP enzyme activity assay

Unfixed cryosections from *Pde6a* mutants were incubated with an Avidin/Biotin Blocking Kit (Vector Laboratories, Burlingame, CA, USA), followed by incubation at 37 °C for 2 h in PARP reaction mixture containing 10 mM MgCl_2_, 1 mM DTT, 5 mM biotinylated NAD^+^ (Trevigen, Gaithersburg, MD, USA) in 100 mM Tris buffer with 0.2% Triton X-100 (pH 8.0). Incorporated biotin was detected by binding to avidin conjugated with Alexa Fluor 488 (1:800, 1 h at room temperature; Life Technologies, Darmstadt, Germany). For controls, biotinylated NAD^+^ was omitted from the reaction mixture (not shown, but see Paquet-Durand *et al*.^10^).

### PAR immunohistochemistry

For the PAR staining, fixed cryosections were dried at 37 °C for 1 h, and washed with PBS for 10 min at room temperature. To reduce nonspecific background, quenching solution (100 *μ*l 30% H_2_O_2_, 400 *μ*l MeOH, 500 *μ*l PBST) was put on each section for 20 min at room temperature. Sections were incubated in blocking solution (10% NGS, 0.1% PBST) for 1 h at room temperature. Primary PAR antibody (1:200; no. ALX-804-220; Enzo Life Sciences, Loerrach, Germany) was applied overnight at 4 °C. After washing three times with PBS for 1 h, sections were incubated with secondary antibody (biotinylated goat anti-mouse 1:150; Vector Laboratories, Burlingame, CA, USA). This was followed by incubation with Vector ABC Kit (Vector Laboratories) for 1 h in ABC reaction mixture (1 *μ*l avidin solution, 1 *μ*l biotin solution, in 148 *μ*l PBS). After washing three times with PBS for 10 min, sections were visualized under a microscope.

### Microscopy, cell counting, and statistical analysis

Light and fluorescence microscopy was performed on a Z1 ApoTome Microscope equipped with a Zeiss Axiocam Digital Camera (Zeiss, Jena, Germany). Images were captured using Zeiss Axiovision 4.7 software (Zeiss) and representative pictures were taken from central areas of the retina. Adobe Photoshop CS3 (Adobe Systems Incorporated, San Jose, CA, USA) and Corel Draw X3 software (Corel Corporation, Ottawa, Canada) was used for image processing.

For cell quantifications, pictures were captured on whole radial sections using the Mosaix mode in Axiovision 4.7. TUNEL, PARP activity, and PAR-labelled cells were counted manually. First, the average size of an ONL cell was determined by counting all DAPI-positive cells in 3–6 rectangular areas distributed randomly over the ONL.^11,14^ This was repeated for retinal sections obtained from five different animals and the ONL cell size averaged. The total number of cells was then determined by dividing ONL area through the average ONL cell size. The number of positive cells was then divided by the total number of ONL cells giving the percentage of positive cells. All data given represent the means and standard deviation from three sections each for each animal, obtained from at least three different animals.

Statistical comparisons between experimental groups were made using one-way ANOVA and Bonferroni’s correction using Prism 5 for Windows (GraphPad Software, La Jolla, CA, USA). Values are given as mean±S.E.M. Levels of significance were: not significant (NS)=*P*>0.05, *=*P*<0.05, **=*P*<0.01, and ***=*P*<0.001.

## Abbreviations

DIV: days *in vitro*
GCL: ganglion cell layer
INL: inner nuclear layer
PDE: phosphodiesterase
ONL: outer nuclear layer
P: postnatal day(s)
PARP: poly(ADP-ribose)polymerase
PAR: poly(ADP-ribose)
RP: retinitis pigmentosa
TUNEL: terminal deoxynucleotidyl transferase dUTP nick-end labelling.
