# Supplementary figures and images for: Efficacy of PARP inhibition in Pde6a mutant mouse models for retinitis pigmentosa depends on the quality and composition of individual human mutations
Source: Cell Death Discov. 2016 Jul 4;2:16040–. doi: 10.1038/cddiscovery.2016.40 (PMC4979439; doi:10.1038/cddiscovery.2016.40)

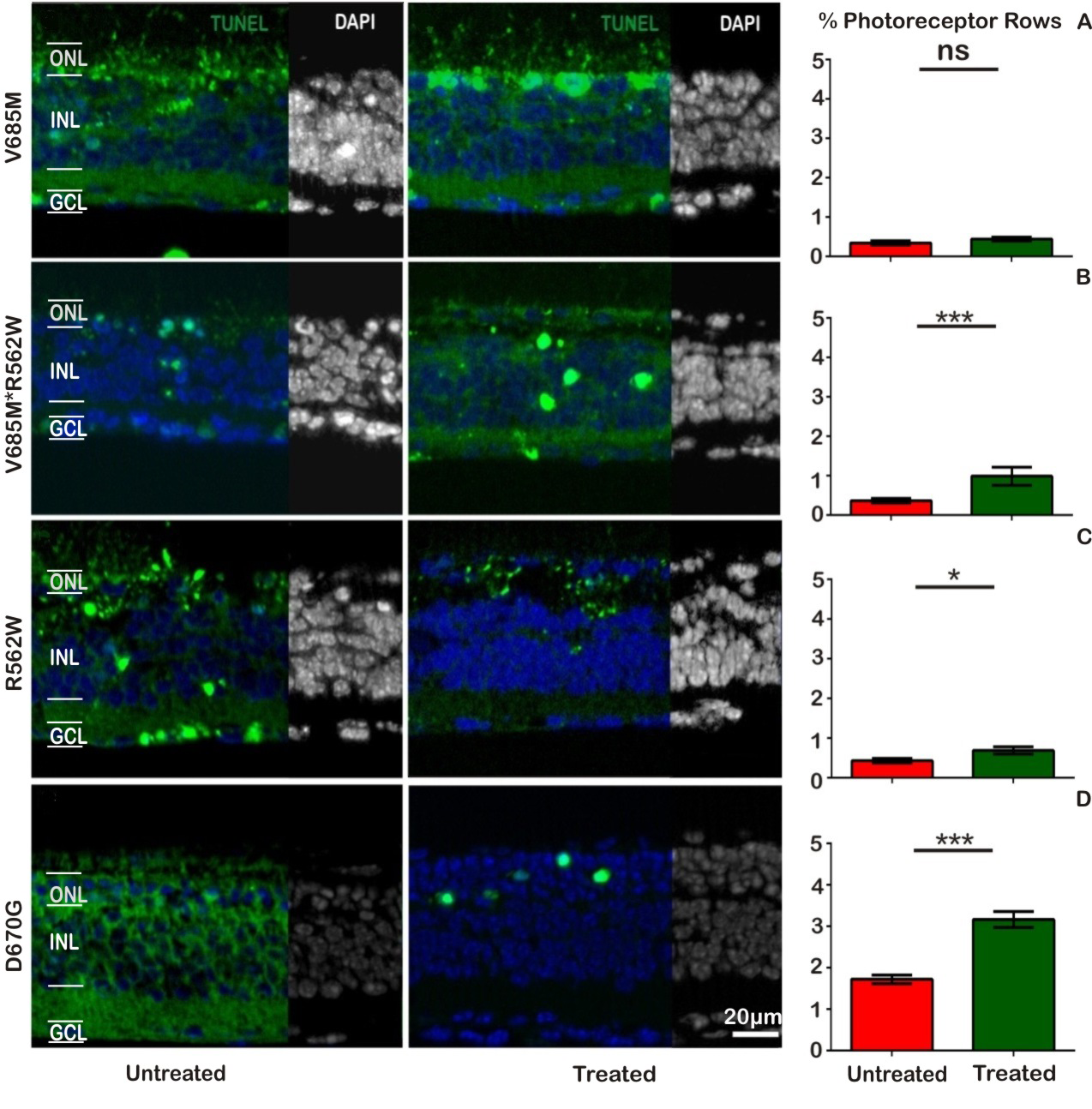

Supplement: Supplementary Figure 1 [file cddiscovery201640-s1.tiff]

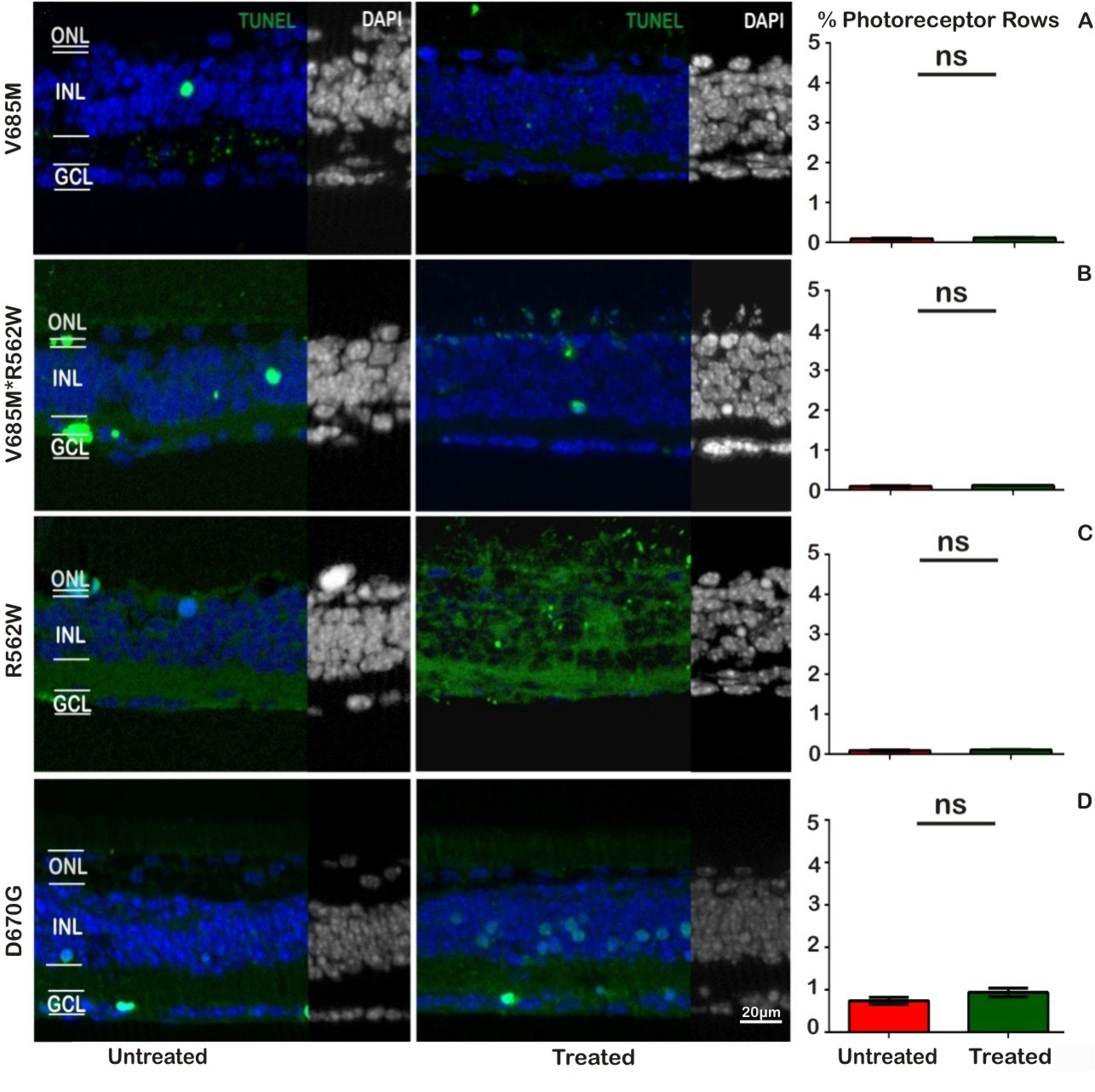

Supplement: Supplementary Figure 2 [file cddiscovery201640-s2.tiff]
